# Pupillary Responses to Dot Patterns on a Human Face Background

**DOI:** 10.3390/bs14010069

**Published:** 2024-01-19

**Authors:** Nanxin Song, Shinichi Koyama

**Affiliations:** Institute of Art and Design, University of Tsukuba, Ibaraki 305-8574, Japan; s1930516@s.tsukuba.ac.jp

**Keywords:** trypophobia, disgust, dot patterns, HASU-COLLA, pupillometry

## Abstract

Dots on natural backgrounds can elicit significant pupillary constrictions within the entire image phase associated with parasympathetic activation, suggesting disgust rather than fear. Although studies have reported that dots on faces elicit stronger disgust than dots on non-face backgrounds, it remains unclear whether dots on a face elicit stronger pupil constrictions than non-face backgrounds. Pupillometry was used while viewing dots on faces and compared with luminance- and spatial frequency-controlled images (dots on phase-scrambled faces) and luminance-controlled images (face only, phase-scrambled faces). Relative pupillary constrictions were elicited when dots were placed on faces and phase-scrambled faces; however, the response to dots on faces did not differ significantly from that to the control stimuli. Approximately 3–5 s after stimulus onset, pupillary responses to dots on faces recovered to baseline faster than those to dots on phase-scrambled faces with a larger pupil size. The initial pupillary constrictions observed are consistent with those in response to dots on natural backgrounds, suggesting that regardless of the background, dots may stimulate parasympathetic activation and elicit disgust rather than fear. The faster recovery from the pupil constriction and larger pupil size in the later phase may be caused by a dynamic balance between the sympathetic and parasympathetic neuronal activities.

## 1. Introduction

Dot patterns are widely applied in media and visual environments and can cause discomfort for viewers [1,2]. This discomfort is referred to as trypophobia [3] and is considered to elicit disgust rather than fear. A study by Ayzenberg et al. [4] demonstrated that sustained significant pupillary constrictions occurred in the observation of dot patterns on natural backgrounds within 5 s of the image phase (i.e., trypophobic images [3]). Pupillary constrictions are thought to be stimulated by the parasympathetic nervous system [5,6,7,8] and are associated with disgust rather than fear [9,10,11,12,13,14]. Conversely, it has also been reported that trypophobic images stimulate the sympathetic nervous system in ways such as an increased heart rate in individuals with trypophobia [15].

Interestingly, strong disgust can be elicited when dot patterns are present on human skin and faces [16,17,18]. Dot patterns on human skin and faces have been referred to as HASU-COLLA (HASU = lotus seed pods; COLLA [i.e., collage] = photomontage image) and are used by motives by Internet artists in Japan [19]. This strong sense of disgust has been explained by contrasting energies in spatial-frequency characteristics [3,20,21] and the perceived spatial relationship between dots and human face backgrounds that can trigger memories of skin diseases [22,23,24,25,26,27].

While it is known that images of dot patterns on a human face background can elicit strong disgust, it is less clear whether they can elicit sustained, significant pupillary constrictions, such as those seen with dot patterns on natural backgrounds [4] (i.e., non-HASU-COLLA trypophobic images [3]), throughout the entire image phase caused by the activation of the parasympathetic nervous system. Therefore, this study investigated how pupil size changes when observing dots on the human face background during the entire image phase and whether dots on the face activate the parasympathetic nervous system with significantly sustained pupillary constrictions and a strong reaction of disgust rather than one of fear. Following a previous study [4], we compared changes in pupil size during the observation of images of dots on a face as the target stimuli (i.e., HASU-COLLA images), images of dots on a non-face (i.e., non-HASU-COLLA trypophobic images [3,4]), images of a face only, and images of non-dots and non-faces as control stimuli. As we tend to feel stronger disgust toward dot patterns when they are presented on human faces [16,17,18] rather than on other backgrounds, it was predicted that we would observe greater pupil constriction for dots on the face than for those on non-faces. To enable comparisons across stimuli and avoid explanations based on luminance and spectrum differences [28,29], we created luminance- and spatial-frequency-controlled images (dots on the phase-scrambled face, the face only, and the phase-scrambled face).

## 2. Materials and Methods

### 2.1. Participants

We chose the sample size to enable the detection of a medium effect size (*α* = 0.05, 1 − *β* = 0.80, f = 0.5) in a within-participant factor. The use of G*power [30] suggested 34 participants were needed. Thus, 34 undergraduate and graduate students from the University of Tsukuba participated in this study (mean age = 24.4 years; SD = 0.5; range = 18–31 years; 23 females and 11 males). None of the participants reported a history of neurological disease; head injury; alcohol, caffeine, and cigarette abuse; sleep disorders; or strenuous physical exercise before the experimental session. They had normal or corrected-to-normal vision.

### 2.2. Stimuli

**Figure 1:**
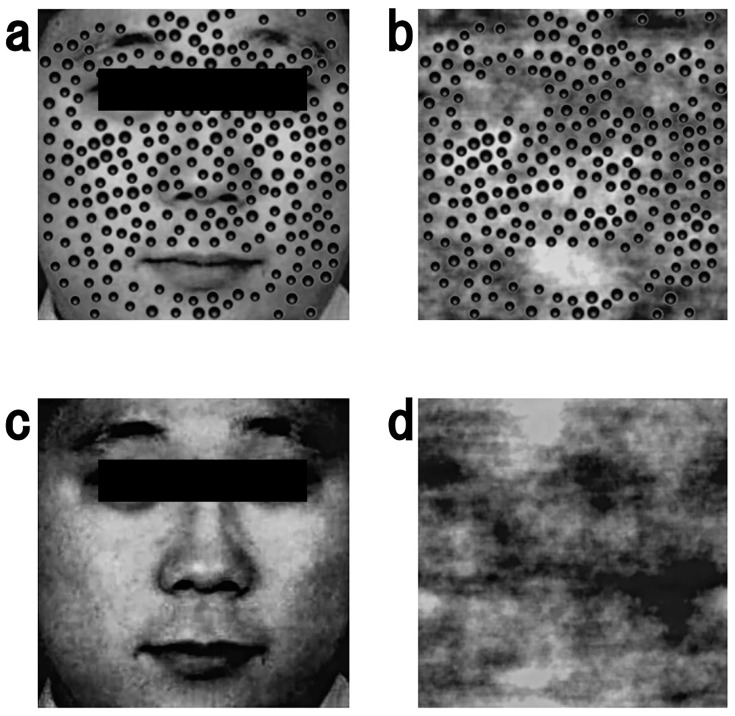
Examples of stimuli with black rectangles. Note that in the actual experiment, stimuli were presented without black rectangles: (**a**) dots on the face, (**b**) dots on the phase-scrambled face, (**c**) face-only images, (**d**) phase-scrambled face. Luminance of all stimuli was matched, and the average luminance of the stimuli was 33.0 cd/m^2^. Permission for JACNeuF images [31] was obtained from Humintell/David Matsumoto.

The stimuli consisted of eight frontal facial images of four Japanese females and four Japanese males. Two female and two male facial images were obtained from Japanese and Caucasian neutral faces (JACNeuF) [31], and two female and two male facial images were obtained from the Sozai-jiten Picture Database [32]. We converted these eight facial images into grayscale (512 × 512 pixels; 13.5° × 13.5° visual angle) and eight phase-scrambled facial images using MATLAB. Next, we added the same cluster of 245 dot patterns to the eight facial images and eight phase-scrambled facial images, excluding the eyebrows, eyes, hair, and mouth, to create stimuli with dots following the skin disease hypothesis [22,23,24,25,26,27]. Among the clusters of dot patterns, 73 dot pattern sizes were 0.3° in diameter, and the remaining 172 dot pattern sizes were 0.4° in diameter [17]. After adding clusters of dot patterns, we created four types of stimuli (see Figure 1a–d): eight images of dots on the face, eight images of dots on the phase-scrambled face, eight images of the face only, and eight images of the phase-scrambled face. All the images were centered on a uniform gray background (1920 × 1080 pixels, mean luminance = 33.0 cd/m^2^). We matched the luminance among the stimuli using the SHINE toolbox in MATLAB [33]. Finally, the average luminance of all the stimuli was 33.0 cd/m^2^.

In order to ensure the congruence of spatial-frequency spectra across various conditions, we matched the spatial-frequency spectrum between dots on the face and those on the phase-scrambled face images, as well as the face-only and phase-scrambled face-only images. Moreover, we nearly matched the spatial-frequency spectra for all four types of stimuli. Subsequently, an analysis of the spatial-frequency spectra for these four stimulus types was conducted using the SHINE toolbox. In the lower range (10–60 cycles per image), the contrast energy of the dots on the face was significantly higher than that of dots on the phase-scrambled face (*p* < 0.05). By contrast, in the higher range (64–65, 68, 86–88, 90–93, 95–98, 125, and 127–128 cycles per image), the contrast energy of dots on the phase-scrambled face was significantly higher than that of dots on the face (*p* < 0.05). However, there were no significant differences in the contrast energies of face-only and phase-scrambled face-only images at any spatial frequency. In total, 32 stimulus images were presented on a 23-inch Tobii Pro TX300 eye-tracker screen (1920 × 1080 pixels, 300 Hz; Tobii Technology, Stockholm, Sweden).

### 2.3. Apparatus

We conducted the experiment in a lit room without windows (approximately 1000 lx. We used a 23-inch Tobii screen as the experimental apparatus to present all images (1920 × 1080 pixels, Tobii Technology, Stockholm, Sweden). We recorded changes in pupil size using a Tobii TX300 infrared binocular eye tracker at a rate of 300 Hz. We used a chinrest to minimize the participants’ body movements and keep their gaze directed toward the screen while observing the stimuli.

### 2.4. Procedure

Participants were instructed to rest on a comfortable chair and fix their heads on a chinrest. At the beginning of the experiment, they focused on a fixation cross in the middle of the Tobii screen. The viewing distance was fixed at 60 cm from the display. The fixation cross “+” was presented at the center of the gray background of the screen. It subtended approximately 1.0° horizontally and 1.0° vertically. Next, all participants’ gazes were calibrated using a 9-point calibration routine. After calibration, 32 main trials were conducted. Each trial began with a series of instructions, and then a uniform gray background image was displayed (mean luminance = 33.0 cd/m^2^) for 1 min to adapt the pupil sizes so that the change in their pupil sizes could be used as the baseline. Each of the 32 images (eight images of dots on the face, eight images of dots on the phase-scrambled face, eight images of the face only, and eight images of the phase-scrambled face) was presented randomly for 6 s.

## 3. Results

We analyzed all participants’ data based on the screening criteria for the quality of pupillary response (i.e., higher than 70%) [34]). We examined each participant’s mean percentage change in pupil size from their baselines. We determined each baseline pupil size as the average pupil size during the 1000 ms preceding the onset of each stimulus for each participant. The presentation of stimuli resulted in a change in pupil size relative to the baseline (i.e., 0%), and the stimuli evoked a smaller pupil size relative to the baseline (i.e., pupil constriction), resulting in a negative percentage change corresponding to a reduction in pupil size. On the other hand, the stimuli evoked a large pupil size relative to the baseline (i.e., pupil dilation), resulting in a positive percentage change corresponding to an increase in pupil size. 

**Figure 2:**
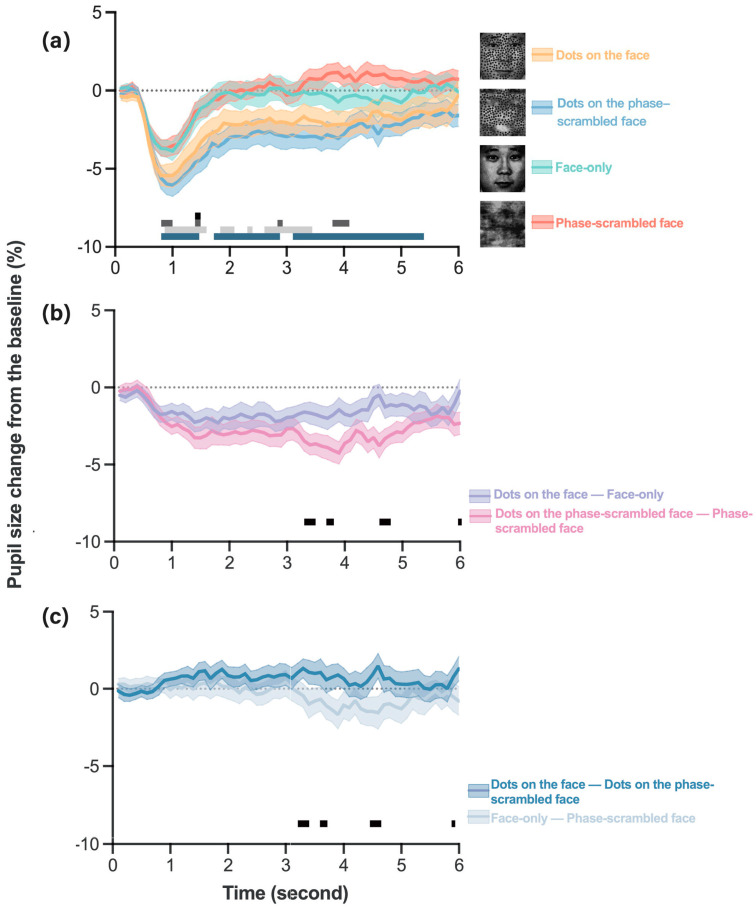
Percentage changes of pupil size from baseline. The *x*-axis represents trial time in seconds (s), and the *y*-axis represents the percentage change (in seconds) of pupil size relative to the baseline. A negative percentage indicates pupil constriction, whereas a positive percentage indicates pupil dilation. Colored shadows represent the standard error of the mean (SEM). (**a**) Pupillary response across time for each stimulus type. A black sold line represents greater reduction in pupil size to dots on the face than to the face only during 1.4 s (*p*s < 0.01). A dark gray sold line represents a greater reduction in pupil size to dots on the face than to the phase-scrambled face during 0.8–1.0 s, 1.4 s, 2.8 s, and 3.7–4.1 s (*p*s < 0.01). A light gray solid line represents a greater reduction in pupil size to dots on the phase-scrambled face than to the face only at 0.9–1.6 s, 1.8–2.1 s, 2.3–2.4 s, and 2.6–3.6 s (*p*s < 0.01). A blue-gray solid line represents a greater reduction in pupil size in response to dots on the phase-scrambled face than in response to the phase-scrambled face at 0.8–1.6 s, 1.8–2.9 s, 3.1–5.4 s (*p*s < 0.01). (**b**) Pupillary responses across time for the difference between dots on the face and face-only images and the difference between dots on the phase-scrambled face and the phase-scrambled face. A black solid line represents a greater reduction in pupil size in response to dots on the phase-scrambled face − phase-scrambled face than those in response to dots on the face − face-only images during 3.3–3.5 s, 3.8–3.9 s, 4.6–4.8 s, and 6.0 s (*p*s < 0.05). (**c**) Pupillary responses across time for the difference between dots on the face and dots on the phase-scrambled face and the difference between the face only and the phase-scrambled face. A black solid line represents a greater reduction in pupil size in response to the face only − phase-scrambled face than those in response to dots on the face − dots on the phase-scrambled face during 3.3–3.5 s, 3.8–3.9 s, 4.5–4.8 s, and 6.0 s (*p*s < 0.05). Permission for JACNeuF images [31] was obtained from Humintell/David Matsumoto.

To demonstrate the effect of stimulus type on pupillary responses, we conducted a repeated-measures analysis of variance (ANOVA), with percentage of pupil size change as the dependent variable. The results showed a significant main effect of stimulus type, *F* (3, 99) = 11.66, *p* < 0.01, *η^2^* = 0.22 (Figure 2a). Bonferroni adjustment tests showed no significant difference in pupil size change between dots on the face and dots on the phase-scrambled face (*p* = 1.0). Significant pupillary constriction in response to dots on the face was observed compared to face-only (*p* < 0.01) and phase-scrambled face images (*p* < 0.01). Results also showed that significant pupillary constriction in response to dots on the phase-scrambled face was observed compared to face-only (*p* < 0.01) and phase-scrambled face images (*p* < 0.01). 

Given the significant interaction effect of stimulus type and time, *F* (177, 5841) = 2.45, *p* < 0.01, *η^2^* = 0.07, we conducted simple effects of stimulus type at each time point. These analyses revealed that reductions in pupil size in response to dots on the face were significantly greater than those in response to face-only images at 1.4 s (*p* < 0.01, dots on the face < 0, face only < 0) and phase-scrambled face images at 0.8–1.0 s, 1.4 s, 2.8 s, and 3.7–4.1 s (*p*s < 0.01, dots on the face < 0, phase-scrambled face < 0 at 0.8 s–1.0 s, 1.4 s, phase-scrambled face > 0 at 3.7–4.1 s). Reductions in pupil size in response to dots on the phase-scrambled face were significantly greater than those in response to the face only at 0.9–1.6 s, 1.8–2.1 s, 2.3–2.4 s, 2.6–3.6 s (*p*s < 0.01, dots on the phase-scrambled face < 0, face only < 0) and phase-scrambled face at 0.8–1.6 s, 1.8–2.9 s, 3.1–5.4 s (*p*s < 0.01, dots on the phase-scrambled face < 0, phase-scrambled face < 0 at 0.8–1.6 s, 1.8–2.1 s, 2.3 s, 3.1–3.2 s, phase-scrambled face > 0 at 2.2 s, 2.4–2.9 s, 3.3–5.4 s). There were no significant differences in the pupillary responses to dots on the face and those to dots on the phase-scrambled face within 6 s of the image phase.

To assess the dot effect (Figure 2b) for face versus phase-scrambled face background images on pupillary response, we compared the difference in percentage of pupil size change between dots on the face and face-only images versus the difference in the percentage of pupil size change between dots on the phase-scrambled and phase-scrambled face-only images (“dots on the face − face only” vs. “dots on the phase-scrambled face − phase-scrambled face only”). We then conducted a paired-samples *t*-test with differences from subtractions of the percentage of pupil size change for each time point. The paired-samples *t*-test showed no significant difference in the mean pupil size changes between dots on the face − face only vs. dots on the phase-scrambled face − phase-scrambled face (*p* = 0.25). However, the paired-samples *t*-test demonstrated a greater increase in pupil size in response to dots on the face − face-only images than those to dots on phase-scrambled face − phase-scrambled face images at 3.3–3.5 s, 3.8–3.9 s, 4.6–4.8 s, and 6.0 s (*p*s < 0.05, dots on the face − face only < 0, dots on the phase-scrambled face − phase-scrambled face-only images < 0).

To assess the amplified face background effect in images with versus those without dots (Figure 2c) on pupillary response, we compared the difference in the percentage of pupil size change between dots on the face and dots on the phase-scrambled face versus that between face-only and phase-scrambled face-only images (“dots on the face − dots on the phase-scrambled face” vs. “face only − phase-scrambled face”). Subsequently, we conducted a paired-samples *t*-test with differences from subtractions of the percentage of pupil size change for each time point. There was no significant difference in the mean pupil size changes between dots on the face − dots on the phase-scrambled face versus face-only − phase-scrambled face images (*p* = 0.25). However, the paired-samples *t*-test demonstrated a greater reduction in pupil size in response to dots on face—dots on phase-scrambled face than in response to face only − phase-scrambled face at 3.3–3.5 s, 3.8–3.9 s, 4.5–4.8 s, and 6.0 s (*p*s < 0.05, dots on the face − dots on the phase-scrambled face > 0, face-only − phase-scrambled face images < 0).

## 4. Discussion

The present study showed relative pupillary constrictions in response to dots on the face and dots on the phase-scrambled face compared with those in response to face-only and phase-scrambled face images. These pupillary constrictions are consistent with those in response to dots on the natural backgrounds, as found by Ayzenberg et al. [4]. These results suggest that, regardless of the background image, dot patterns can stimulate the parasympathetic nervous system [5,6,7,8] and elicit disgust rather than fear [9,10,11,12,13,14,16,17,18].

However, contrary to our hypothesis, we did not observe greater pupil constriction in response to the dots on the face compared with those on the scrambled faces in the present study. Although previous studies have shown that there are neuronal mechanisms specialized for the processing of faces [35,36], and that dot patterns elicit stronger disgust when they are perceived as part of a face [16,17,18], we did not observe any face-specific pupil constriction effect in the present study. Possible explanations for the similar results between dots on the face versus those on the scrambled face are that (1) there might be distorted face-like patterns in some images of phase-scrambled faces, which elicited an even greater pupil constriction or that (2) phase-scrambled images per se caused strong disgust and pupil constriction.

By contrast, approximately 3–5 s after stimulus onset, pupillary constriction recovered faster with dots on the face than with dots on the scrambled face (Figure 2b,c), demonstrating that having a face as the background image may facilitate recovery from pupillary constriction. Studies have shown that images containing faces may activate the sympathetic nervous system [37,38], which may elicit pupil dilation [39,40].

This study has some limitations. First, the spatial frequency spectrum was only nearly matched between the images with and without dots, which may have had a minor impact on pupillary responses. Second, our results may differ for individuals with different sensory characteristics, such as those with trypophobia. The current study provides observational data indicating pupillary responses; however, further research is required to investigate the gap between emotional experiences and physiological responses.

## 5. Conclusions

The current study provides observational data indicating that pupils exhibited relative constriction in response to dots on the face and dots on the phase-scrambled face compared with those in response to face-only and phase-scrambled face images. Such pupillary constriction is aligned with that observed in response to dots on natural backgrounds (i.e., trypophobic images), suggesting that, regardless of the background image, dot patterns can stimulate parasympathetic activation, causing pupillary constriction accompanied by a sense of disgust rather than one of fear. Conversely, approximately 3–5 s after onset, the pupillary response to dots on the face recovered to baseline faster than that in response to the control stimuli (dots on the phase-scrambled face), with a larger pupil size. These dynamic changes in pupillary size suggest that dots on the face may engage complex neural mechanisms, such as opposing functions of the parasympathetic and sympathetic nervous systems.

## Data Availability

The data supporting the findings of this study are available from the corresponding author upon reasonable request.
